# Supplementary figures and images for: Identification of kinases activated by multiple pro-angiogenic growth factors
Source: Front Pharmacol. 2023 Jan 4;13:1022722. doi: 10.3389/fphar.2022.1022722 (PMC9847502; doi:10.3389/fphar.2022.1022722)

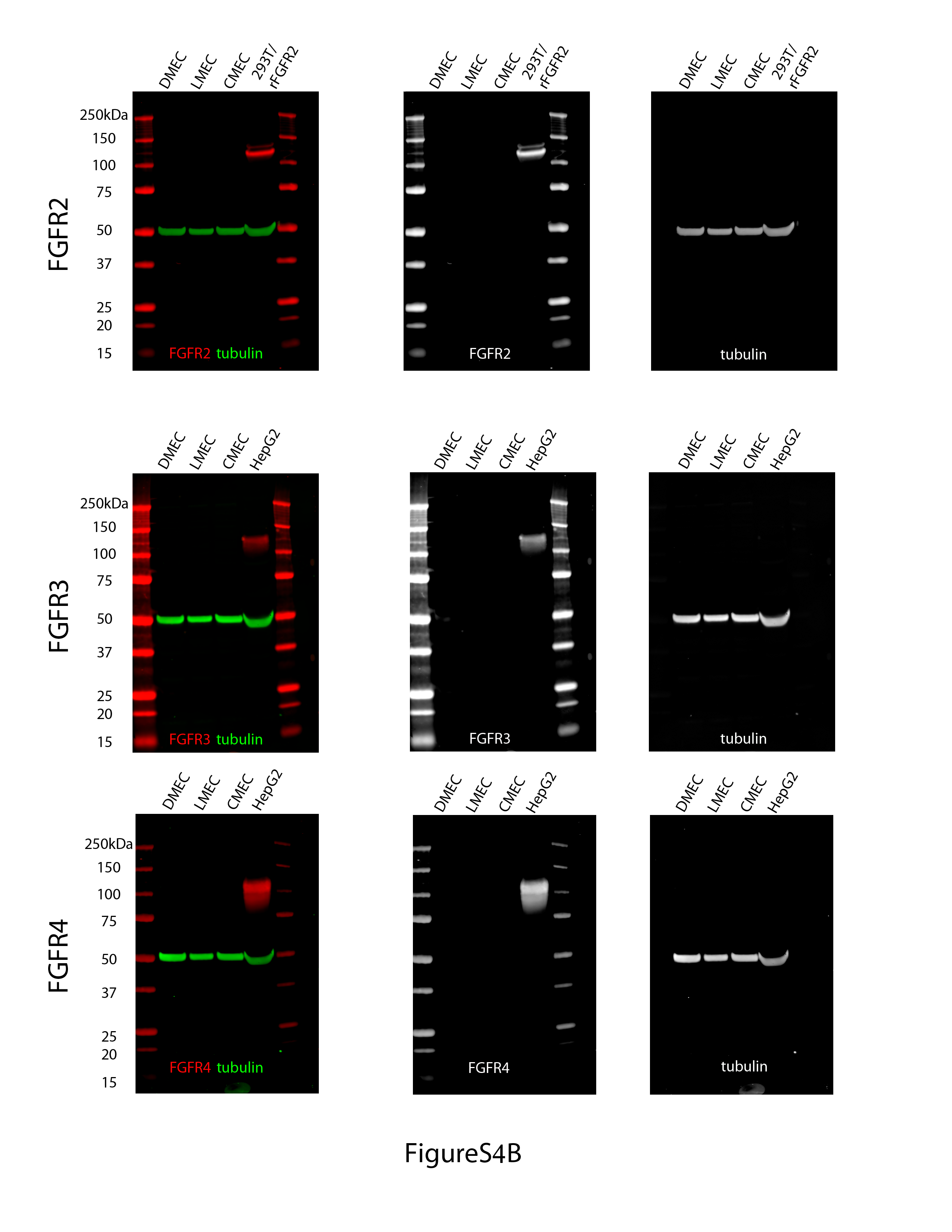

Supplement: Supplementary file 1 [file Image5.PNG]

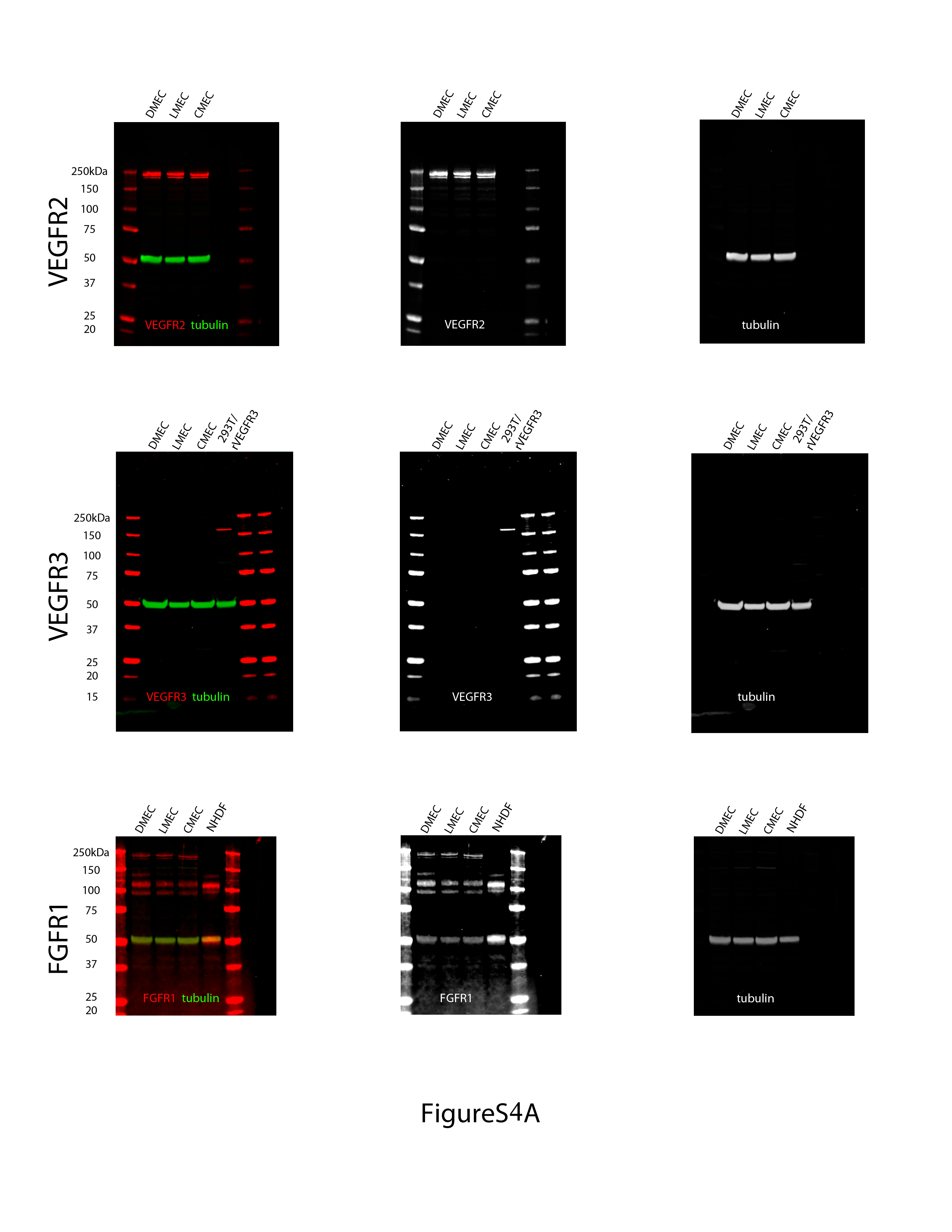

Supplement: Supplementary file 2 [file Image4.PNG]

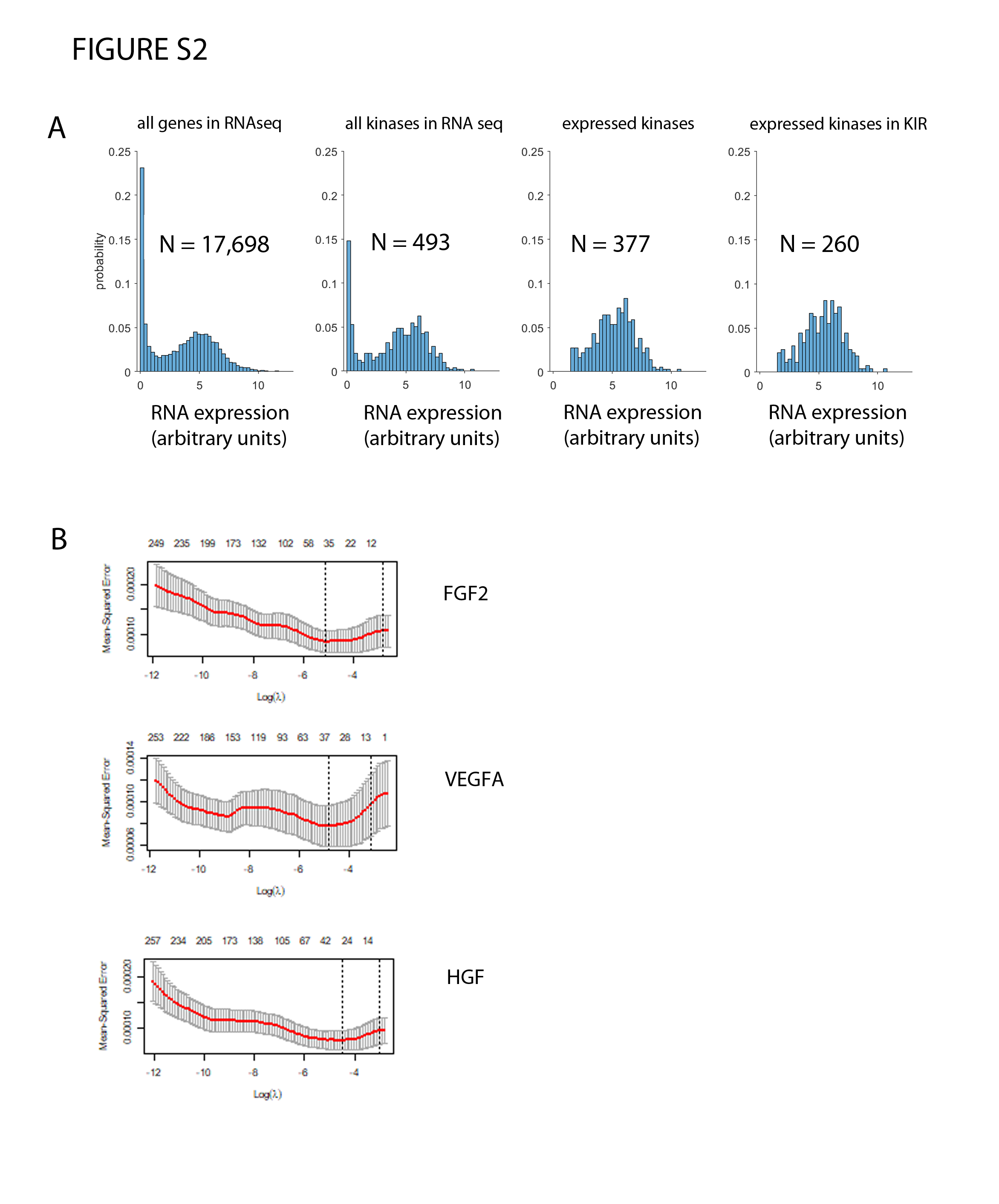

Supplement: Supplementary file 3 [file Image2.PNG]

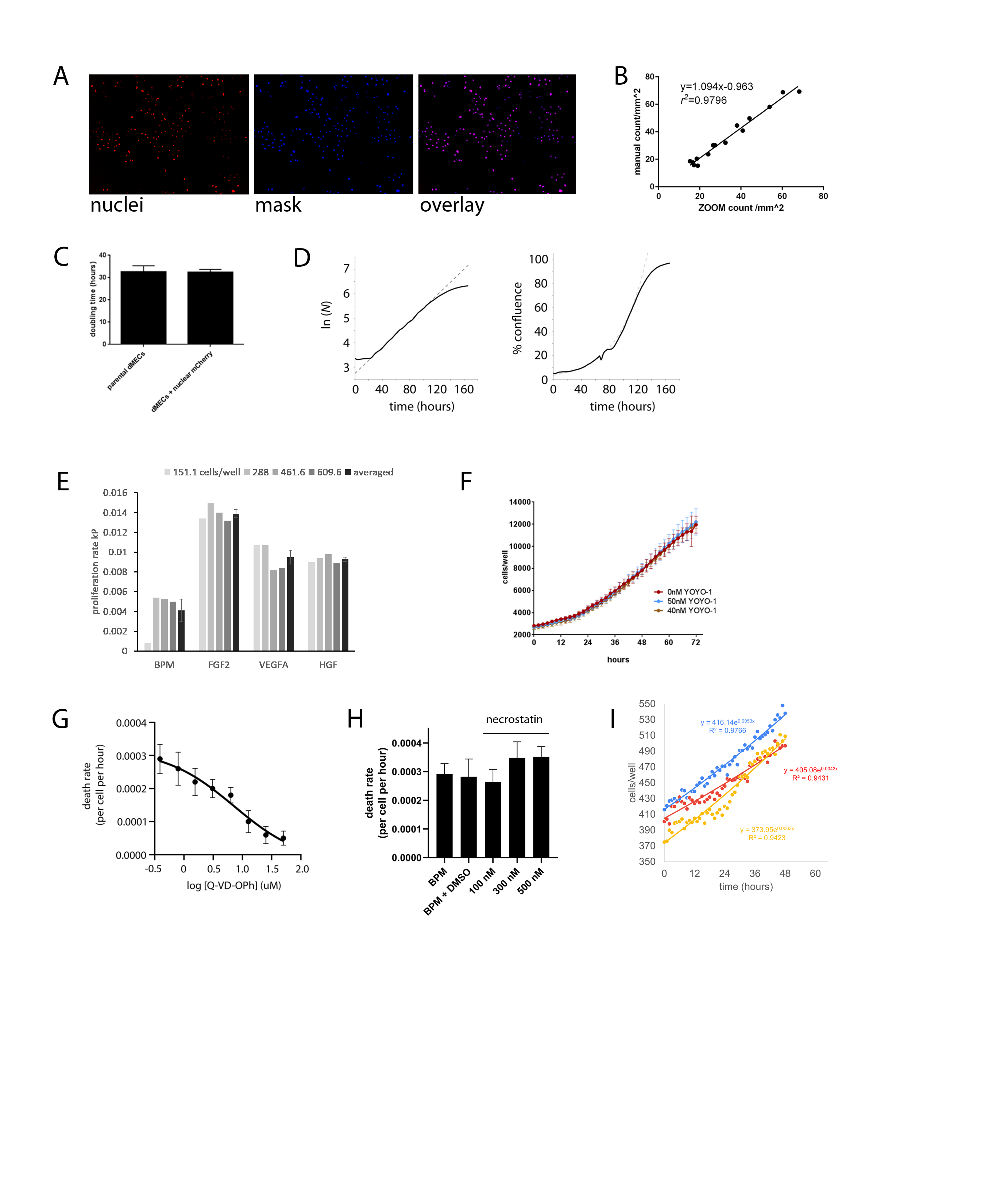

Supplement: Supplementary file 4 [file Image1.PNG]

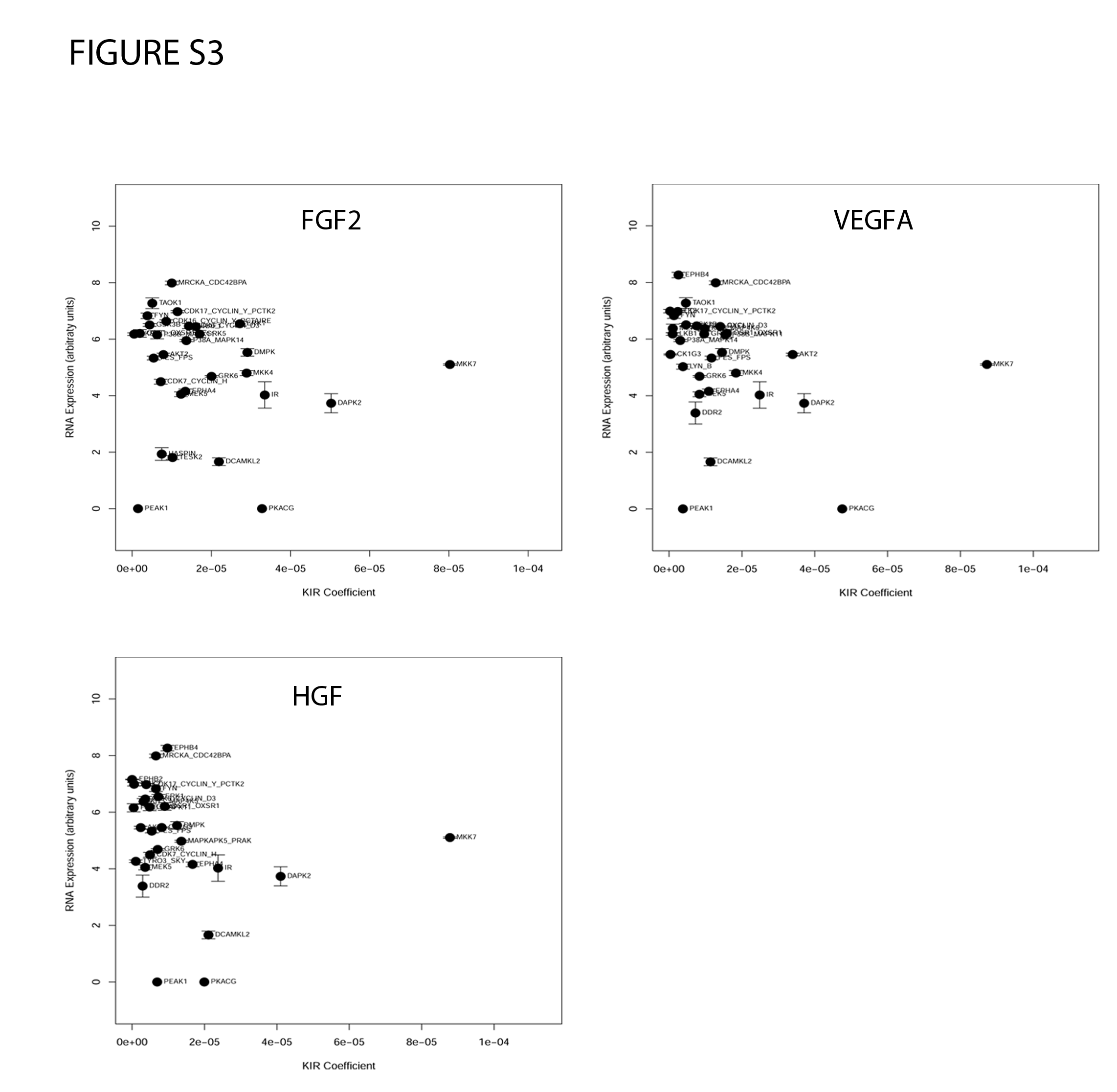

Supplement: Supplementary file 5 [file Image3.PNG]
